# Schulische Gesundheitsförderung und Prävention in Deutschland. Aktuelle Themen, Umsetzung und Herausforderungen

**DOI:** 10.1007/s00103-022-03558-3

**Published:** 2022-06-24

**Authors:** Kevin Dadaczynski, Orkan Okan, Freia De Bock, Uwe Koch-Gromus

**Affiliations:** 1 Fachbereich Gesundheitswissenschaften, Hochschule Fulda, Leipziger Str. 123, 36037 Fulda, Deutschland; 2 Zentrum für Angewandte Gesundheitswissenschaften, Leuphana Universität Lüneburg, Wilschenbrucher Weg 84A, 21335 Lüneburg, Deutschland; 3 Fakultät für Sport- und Gesundheitswissenschaften, Technische Universität München, Georg-Brauchle-Ring 60/62, 80992 München, Deutschland; 4 Bereich Versorgungsforschung im Kindes- und Jugendalter, Klinik für Allgemeine Pädiatrie, Neonatologie und Kinderkardiologie, Universitätsklinikum Düsseldorf, Moorenstr. 5, 40225 Düsseldorf, Deutschland; 5 Institut und Poliklinik für Medizinische Psychologie, Universitätsklinikum Hamburg-Eppendorf, Martinistr. 52, 10246 Hamburg, Deutschland

Im Schuljahr 2020/2021 besuchten 8,35 Mio. Kinder und Jugendliche eine von mehr als 31.000 allgemeinbildenden Schulen in Deutschland. Zugleich ist das allgemeinbildendende Schulsystem Arbeitgeber von etwa 700.000 in Vollzeit und Teilzeit tätigen Lehrkräften [1], wobei das weitere Schulpersonal (z. B. Schulsozialarbeiter*innen, -psycholog*innen, Inklusionshelfer*innen) noch hinzuzurechnen ist. Nicht zuletzt aufgrund der Relevanz, die der Bildungsort Schule für alle dort Tätigen und Lernenden hat, hat sich die Schule seit Verabschiedung der Ottawa-Charta im Jahr 1986 weltweit zu einem zentralen Setting der Gesundheitsförderung und Prävention entwickelt [2]. Dabei wird die Schule auch als geeigneter Ort gesehen, alle Kinder und Jugendlichen ungeachtet ihrer sozialen, kulturellen oder religiösen Herkunft zu erreichen und mit gesundheitsbezogenen Maßnahmen anzusprechen. Hierüber soll ausdrücklich auch ein Beitrag zur Verbesserung der Bildungsqualität geleistet werden [3]. Derzeit existiert eine Vielfalt von Ansätzen, die unterschiedliche Präventionsebenen (Verhalten, Verhältnisse), Zielgruppen (Schüler*innen, Lehrkräfte, Eltern) und Themen (z. B. körperliche Aktivität, psychische Gesundheit, Ernährung, gesundheitsförderliche Organisationsentwicklung) adressieren. Diese werden auf Ebene von Kommune, Land und Bund mit unterschiedlicher Komplexität und Reichweite umgesetzt [4]. International besonders favorisiert (z. B. durch die Weltgesundheitsorganisation oder das Schools-for-Health-in-Europe-Netzwerk) wird dabei der Ansatz der „gesundheitsfördernden Schule“, der aufgrund seiner Ausrichtung an verschiedenen Zielgruppen, Themenfeldern und Ebenen innerhalb und außerhalb der Schule auch als „whole school approach to health“ (ganzheitlicher Schulansatz für Gesundheit) bezeichnet wird [5, 6].

Trotz der Vielfalt an bestehenden Ansätzen und Maßnahmen ist die schulische Gesundheitsförderung durch zahlreiche und zum Teil bereits länger bestehende Herausforderungen gekennzeichnet. Hierzu zählt die nach wie vor schwach ausgeprägte Evidenzbasis insbesondere ganzheitlicher Ansätze der schulischen Gesundheitsförderung, die u. a. mit dem Fehlen komplexer Evaluationsansätze begründet werden kann oder auch damit, dass vorhandene komplexere Evaluationsansätze nicht ausreichend angewendet werden [7]. Zum anderen mangelt es abseits der von den gesetzlichen Krankenversicherungen im Rahmen des Gesetzes zur Stärkung der Gesundheitsförderung und der Prävention (Präventionsgesetz – PrävG) realisierten Aktivitäten an abgesicherten Erkenntnissen zum Umsetzungsstand der schulischen Gesundheitsförderung auf Akteurs- und Schulebene in Deutschland [8]. Die schulische Gesundheitsförderung steht in diesem Zusammenhang auch vor der Schwierigkeit, das Thema Gesundheit in einem Setting zu etablieren, welches vor allem durch den Bildungsauftrag legitimiert ist. Dies führt mitunter zu einer selektiven Umsetzung insbesondere in Schulen mit besonderem Interesse an einer Profilbildung im Bereich Gesundheit.

Neben den genannten Schwierigkeiten haben sich infolge der COVID-19-Pandemie auch neue drängende Herausforderungen ergeben. So weisen verschiedene Studien auf pandemiebezogene gesundheitliche Belastungen hin, die sich bei Kindern und Jugendlichen u. a. in einer Zunahme psychischer Belastungen [9], einer Reduktion der körperlichen Aktivität [10] und einer Gewichtszunahme [11] äußern. Dem standen auf der anderen Seite Schulschließungen und die Umstellung von Präsenz- auf Fernunterricht im zeitlichen Umfang von insgesamt etwa 38 Wochen gegenüber [12]. Dieses von der UNESCO auch als „Bildungsdisruption“ bezeichnete Problem dürfte mit hoher Wahrscheinlichkeit dazu geführt haben, dass gesundheitsförderliche Maßnahmen nicht in dem gewohnten Umfang umgesetzt werden konnten, wovon vor allem vulnerable Schüler*innen mit erhöhtem Präventionsbedarf besonders betroffen wären.

Vor diesem Hintergrund zielt das vorliegende Themenheft darauf ab, aktuelle Themen der schulischen Gesundheitsförderung aufzugreifen, Aspekte der Umsetzung entsprechender Aktivitäten zu skizzieren und Herausforderungen und mögliche Lösungsansätze zu diskutieren. Das Themenheft umfasst insgesamt 12 Beiträge, die sich literaturbasiert und empirisch mit verschiedenen Facetten der schulischen Gesundheitsförderung beschäftigen.

Zunächst gibt *P. Paulus* einen Überblick über die Entwicklung der schulischen Gesundheitsförderung in Deutschland und betrachtet dabei den Zeitraum der letzten 30 Jahre. Er behandelt das Thema unter den Perspektiven Praxis, Forschung und Politik. Im anschließenden Beitrag setzen sich *I. Moor et al.* mit einem zentralen gesundheitspolitischen Anspruch von Gesundheitsförderung und Prävention auseinander, nämlich der Reduzierung gesundheitlicher Ungleichheit. Dieses Thema hat eine besonders hohe Relevanz im Kindes- und Jugendalter. Der Beitrag gibt einen Überblick über empirische Belege von gesundheitlicher Ungleichheit bei Kindern und Jugendlichen, zeigt aktuelle Forschungsdefizite auf und diskutiert die mögliche Rolle von Gesundheitsförderung bei der Reduzierung von sozialer Ungleichheit.

*K. Dadaczynski et al.* präsentieren in ihrem Beitrag Ergebnisse einer Studie zum Umsetzungsstand der schulischen Gesundheitsförderung während der Pandemie. Sie identifizieren 3 Cluster schulischer Gesundheitsförderung: COVID-19-bezogene Unterstützung für Schüler*innen, gesundheitsförderliche Gestaltung von Lehr‑, Lern- und Arbeitsbedingungen und Realisierung von Prinzipien der gesundheitsfördernden Schule. Die Ergebnisse geben Hinweise darauf, dass Aspekte der Lehr‑, Lern- und Arbeitsbedingungen sowie der Partizipation und die Kooperation mit schulexternen Akteuren seltener umgesetzt werden. *U. H. Bittlingmayer und G. Okcu* kritisieren in ihrem Beitrag, dass in der bisherigen Diskussion der Rolle der schulischen Gesundheitsförderung bildungssoziologische Aspekte (z. B. Reproduktion außerhalb der Schule liegender sozialer Ungleichheiten, Produktion und Reproduktion von Bildungsungleichheiten) nicht hinreichend Berücksichtigung finden. Deshalb fokussieren sie in ihrem Beitrag bildungssoziologische Positionen und normative Rahmungen der schulischen Gesundheitsförderung. Dabei zeigen sie mögliche Widersprüche zwischen den Zielen der Gesundheitsförderung und den aktuellen schulischen Bedingungen auf.

Neben den Schüler*innen stellt auch das Schulpersonal eine wichtige Zielgruppe der schulischen Gesundheitsförderung dar. Dass Lehrkräfte und Schulleitungen unter der COVID-19-Pandemie im erheblichen Maße beeinträchtigt worden sein könnten, darauf deuten die Ergebnisse der Studie von *J. Hansen et al.* hin. Mit 57,1 % berichten mehr als die Hälfte der befragten Lehrkräfte und Schulleitungen über häufigere Erschöpfungssymptome und 20,6 % über eine verringerte Berufszufriedenheit. Dabei erweisen sich u. a. zusätzliche Arbeitsbelastungen durch die Pandemie, die Belastung durch Unsicherheit sowie auch Sorgen um Schüler*innen als Risikofaktoren der Erschöpfung und der verringerten Berufszufriedenheit.

Die beiden nachfolgenden Beiträge stellen Gesundheitskompetenz (GK) als neues Thema der schulischen Gesundheitsförderung in den Mittelpunkt ihrer Betrachtungen. Gesundheitskompetenz gilt heute als wichtige Ressource. Schulen sind bedeutsame Wirkstätten im Leben junger Menschen und können maßgeblich zur Stärkung von GK beitragen. Im Beitrag von *S. Kirchhoff und O. Okan* werden auf der Basis von Erfahrungen im Projekt „Gesundheitskompetente Organisation Schule“ (GeKoOrg-Schule) Standards herausgearbeitet, die geeignet sind, eine Schule zu einer gesundheitskompetenten Organisation zu entwickeln. Solche Schulen helfen Schüler*innen und dem Personal gezielt, die GK zu stärken. Im nachfolgenden Artikel von *K. Dadaczynski et al.* geht es um die digitale GK von Schüler*innen der Sekundarstufe 1. Dabei berichten 15,3–37,5 % der Befragten von Schwierigkeiten bei der Beschaffung von und im Umgang mit digitalen Informationen, wobei Jugendliche mit niedrigem subjektiven Sozialstatus häufiger eine geringere digitale GK aufweisen. Auch lassen sich verschiedene Zusammenhänge zwischen einzelnen Dimensionen der digitalen GK und dem Ernährungs- und Bewegungsverhalten absichern.

Der abschließende thematische Block bezieht sich auf wichtige Themen und Programme der schulischen Gesundheitsförderung, wobei diese zum Teil eine Good-Practice-Funktion haben. Das von *U. Koller et al.* dargestellte Programm „Fit in Gesundheitsfragen“ bezieht sich auf die Stärkung der Gesundheitskompetenz durch innovative Fortbildungsformate für Lehrkräfte und Unterrichtsmaterialien für Lernende. Dies wird am Modell der Volkskrankheiten Krebs und Diabetes mellitus erprobt. Ergebnisse der begleitenden Evaluation unterstützen den verfolgten Ansatz. *L. Prade et al.* werten in ihrer Untersuchung unter quantitativer wie qualitativer Perspektive Lehrpläne zur Ernährungsbildung (EB) in weiterführenden allgemeinbildenden Schulen aus. Der Zeitumfang und die praktische Umsetzung für die EB erweist sich als stark von schulinternen Faktoren abhängig. Die Autor*innen sprechen sich für eine intensivierte EB in höheren Jahrgängen, die Einführung von Hauswirtschaftsfächern in allen Schulformen und eine bundesweite Vereinheitlichung der Lehrplaninhalte aus.

*C. Stock et al.* richten ihre Aufmerksamkeit auf den riskanten Alkoholkonsum von Jugendlichen. Sie sehen ein großes Potenzial in interaktiven und geschlechtssensiblen schulischen Primärpräventionsangeboten, da in Schulen eine gute Erreichbarkeit der Zielgruppe gewährleistet ist. Ansätze der „virtuellen Simulation“ lassen sich dabei nach Einschätzung der Autor*innen sehr gut und erfolgreich nutzen, da diese Erfahrungen von risikobehafteten Situationen in sicherer Umgebung ermöglichen. Dabei werden auch Herausforderungen gängiger Wirksamkeitsevaluationen wie der randomisierten kontrollierten Studie thematisiert sowie alternative und ergänzende Evaluationsansätze gefordert.

*J. Josupeit et al.* thematisieren die Relevanz von Netzwerken im Kontext der schulischen Gesundheitsförderung. Die Autor*innen kommen zu dem Schluss, dass Netzwerkarbeit für Schulen im Sinne der Gesundheitsförderung obligatorisch sein sollte. Sie kann dazu beitragen, Bedarfe für Gesundheitsförderung innerhalb der Schule, aber auch Lösungsmöglichkeiten aufzuzeigen. Voraussetzungen für effiziente Netzwerkarbeit sind insbesondere eine hohe Motivation der Akteur*innen, eine gemeinsame Vision, feste Strukturen und Kontinuität sowie entsprechende personelle Ressourcen.

Im abschließenden Beitrag des Themenheftes stellt *M. Opitz* das Landesprogramm „Nachhaltige Entwicklung und Etablierung der *Guten Gesunden Schule* im Bundesland Nordrhein-Westfalen“ vor. Beschrieben werden Programmkonzeption, Programmziele, implementierte Strukturen und Erkenntnisse aus der aktuellen Programmevaluation, aber auch die wichtige Vernetzung mit Kooperationspartner*innen. Dabei stellt das „Landesprogramm Bildung und Gesundheit NRW“ ein Dach für die Umsetzung des Programms dar und geht seinem Präventionsauftrag zur Umsetzung der nationalen Präventionsstrategie gemäß § 20f SGB V im Land Nordrhein-Westfalen nach.

Wir wünschen Ihnen, liebe Leserinnen und Leser, eine anregende Lektüre.

Ihre

Kevin Dadaczynski, Orkan Okan, Freia De Bock, Uwe Koch-Gromus
